# Increasing LAG-3 expression suppresses T-cell function in chronic hepatitis B: A balance between immunity strength and liver injury extent

**DOI:** 10.1097/MD.0000000000005275

**Published:** 2017-01-10

**Authors:** Bo Ye, Xuefen Li, Yuejiao Dong, Yiyin Wang, Li Tian, Sha Lin, Xia Liu, Haishen Kong, Yu Chen

**Affiliations:** Key Laboratory of Clinical In Vitro Diagnostic Techniques of Zhejiang Province, Department of Laboratory Medicine, First Affiliated Hospital, College of Medicine, Zhejiang University, Hangzhou, PR China.

**Keywords:** CD8^+^ T cell, chronic HBV infection, interferon-γ (IFN-γ), lymphocyte activation gene-3 (LAG-3)

## Abstract

Weak or absent virus-specific CD8^+^ T-cell responses to hepatitis B virus (HBV) infection are thought to be responsible for persistent HBV infection. Previous studies have indicated that multiple inhibitory receptors, including lymphocyte activation gene-3 (LAG-3), can suppress the CD8^+^ T-cell response in chronic viral infection. This study aimed to detect LAG-3 expression and to investigate the manner in which the immune response is regulated to balance the strength of the response with the extent of liver injury in chronic HBV infection. The results showed that LAG-3 expression levels were significantly higher in CD8^+^ T cells from chronic hepatitis B patients in the immune-active phase compared with chronic asymptomatic HBV carriers and healthy controls. CD8^+^ T-cell function was suppressed in cells with high LAG-3 expression, and these cells exhibited reduced interferon-γ (IFN-γ) secretion. Furthermore, IFN-γ secretion was restored in CD8^+^ T cells that were treated with a specific antibody to LAG-3. Taken together, liver injury was prominent in the immune-active phase, but suppressing T-cell function could mitigate this damage. Importantly, the inhibitory function of LAG-3 can be blocked using a LAG-3-specific antibody, and this can restore the activity of non-functional T cells.

## 1 Introduction

Chronic hepatitis B virus (HBV) infection is a serious public health challenge. It affects more than 300 million people worldwide and has severe consequences, including cirrhosis and hepatocellular carcinoma. HBV is generally considered a non-cytopathic infection, and HBV infection-related liver injury is thought to be a consequence of a specific immune response to HBV-infected hepatocytes.^[1]^ The natural course of chronic HBV infection can be roughly divided into tolerant, immune-active, and inactive phases. Each phase is associated with immune responses of varying intensity and different clinical pathologies. For example, HBV immunity is weak, and liver injury can be absent during the tolerant phase. In the immune-active phase, the immune response is vigorous, and liver injury is prominent.^[2]^ Stronger immune responses are associated with increasingly severe liver injuries. A strong immune response is desirable to clear HBV infection, but severe liver injury can threaten a patient's life if the immune response is too strong. Although this balance is critically important for patient health, it remains unclear how the strength of the immune response during the immune-active phase is regulated.

T cells are an important component of an effective immune response and can be activated and suppressed by regulatory molecules.^[3]^ Recent studies suggest that the suppression of T cell function can be mediated through the sustained expression of inhibitory immunoreceptors.^[4]^ Cell intrinsic negative regulatory pathways can inhibit T cell activation, proliferation, and cytokine production, leading to viral persistence.^[5–8]^ Examples of negative regulatory pathways include programmed death 1 (PD-1), cytotoxic T lymphocyte antigen-4 (CTLA-4), lymphocyte activation gene-3 (LAG-3, CD223), and CD244.^[5–8]^

LAG-3 is an important T-cell negative costimulatory molecule that belongs to the immunoglobulin superfamily. LAG-3 is primarily expressed on the surface of active T lymphocytes, natural killer cells (NK), and dendritic cells (DC). It has functions in homeostasis maintenance and the regulation of the immune response.^[9]^ Recently, Li et al^[7]^ reported that LAG-3 expression was significantly upregulated in CD8^+^ T cells that had infiltrated the tumor lesions of hepatocellular carcinoma patients. Furthermore, the CD8^+^ T cells with elevated LAG-3 expression showed severe functional defects.^[7]^ Additionally, interactions between LAG-3 and its major ligand, Class II MHC, are involved in regulating dendritic cell function.^[10]^ It has also been reported that LAG-3 can maintain a tolerogenic state in CD8^+^ T cells in vivo.^[11]^

The state of LAG-3 expression and the inhibitory effect of LAG-3 on CD8^+^ T-cell function in chronic hepatitis B (CHB) patients remain unclear. In this study, we investigated whether LAG-3 can regulate the strength of the immune response in CHB patients to mitigate liver injury. Additionally, we sought to understand the relationship between the regulation of immune vigor and the extent of liver injury.

## 2 Materials and methods

### 2.1 Study population

Ninety patients with chronic HBV infection were enrolled at the First Affiliated Hospital of Medical College, Zhejiang University, Hangzhou, China between February of 2013 and June of 2014. The patients were divided into 2 groups that included 80 CHB and 10 chronic asymptomatic HBV carriers (ASCs) according to the American association for the study of liver diseases Practice Guidelines. An additional 18 healthy individuals (HCs) were included in the study as controls (Table 1). Exclusion criteria included patients coinfected with human immunodeficiency virus (HIV), hepatitis A virus, hepatitis C virus, or hepatitis D virus. Patients with other chronic liver diseases related to alcohol, drugs, autoimmune diseases, or congestive heart failure were also excluded. This study was approved by the ethics committee of The First Affiliated Hospital, College of Medicine, Zhejiang University, and it was conducted in accordance with the Declaration of Helsinki. Written informed consent was obtained from each participant prior to the study.

**Table 1:**
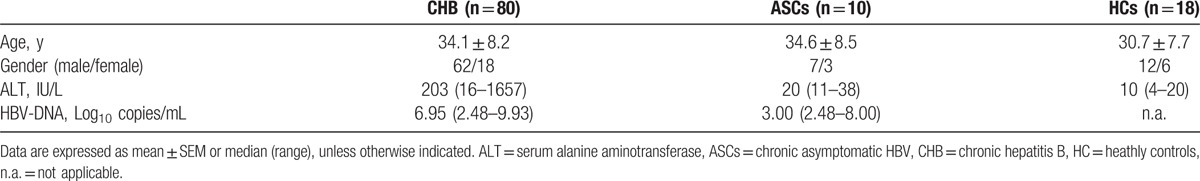
Subject baseline characteristics.

### 2.2 CD223 detection in CD8^+^ T lymphocytes

Peripheral blood mononuclear cells (PBMCs) were separated from whole blood samples using Ficoll-Hypaque centrifugation (Amersham Pharmacia, Uppsala, Sweden). Isolated PBMCs were stained using anti-CD8-fluorescein isothiocyanate (FITC; BD Pharmingen, CA) and CD223-phycoerythrin (PE) mouse anti-human fluorescent monoclonal antibody (R&D Systems, Inc., Minneapolis, MN) for 30 minutes at room temperature. The percentage of CD223 (LAG-3) expression in gated CD8^+^ lymphocytes was detected using a Becton Dickinson FACS system and CELLQuest software (BD Bioscience).

### 2.3 Detection of intracellular IFN-γ

Approximately 2 × 10^5^ PBMCs in RPMI-1640 medium were stimulated using a cell stimulation cocktail (eBioscience, Inc., San Diego, CA) for 6 hours, then collected and stained using FITC conjugated to anti-human CD8 (BD Pharmingen, CA) and APC conjugated to anti-human CD223 (R&D Systems, Inc., Minneapolis, MN). The cells were then washed in washing buffer (included in an IFN-γ ELISPOT kit, Mabtech, OH), and they were fixed and permeabilized. Next, the cells were incubated with PE-conjugated anti-human IFN-γ antibody (BD Pharmingen, CA). IFN-γ-producing cells were identified using an ELISPOT reader (CTL Immunospot, Shaker Heights, OH). Briefly, 96-well plates were precoated with IFN-γ antibodies (Dakewei Biotechnology, Shenzhen, China), and 2 × 10^5^ PBMCs were seeded in the precoated wells and incubated with HBV core antigen (1 μg/mL) with or without 10 μg/mL anti-CD223 antibody for 48 hours at 37 °C and 5% CO_2_. After washing the cells, biotin-conjugated anti-IFN-γ was added to the wells and incubated at room temperature for 1 hour. After incubation with anti-IFN-γ and an additional 5 washes, a horseradish peroxidase-conjugated antibody was added, and the cells were incubated at room temperature for 1 hour. After incubation, unbound antibody was removed by 5 washes. AEC substrates were added and incubated for 30 minutes. Finally, the cells were washed with deionized water 3 to 5 times to stop the reaction. Spot-forming cells (SFC) were counted using an ELISPOT reader.

### 2.4 Detection of HBV virological markers and serum biochemical parameters

Hepatitis B surface antigen (HBsAg), hepatitis B e antigen (HBeAg), hepatitis B surface antibody (anti-HBs), hepatitis B e antibody (anti-HBe), and hepatitis B core antibody (anti-HBc) were detected using a commercial Chemiluminescent Microparticle Immunoassay (CMIA) kit with the Architect-i2000 system (Abbott Laboratories, Abbott Park, IL). Serum HBV-DNA was quantified using the Cobas HBV Amplicor Monitor assay (Roche Diagnostics, Branchburg, NJ) according to the instructions of the manufacturer. The lower HBV DNA detection limit was 2.48 log_10_ copies/mL. Serum alanine aminotransferase (ALT) and aspartate aminotransferase (AST) were detected using a HITACHI 7600–110 auto biochemical analysis system (Hitachi Ltd., Tokyo, Japan), and the normal range of ALT and AST was 5 to 50 IU/L and 8 to 40 IU/L, respectively.

### 2.5 Statistical analysis

GraphPad Prism 5 software was used for all statistical analyses. For patients with undetectable HBV DNA, the lower limit of detection was set at 2.48 log_10_ copies/mL. Quantitative data were expressed as the means ± Standard deviation (SD) or median (range). Differences and correlations between the 2 groups were analyzed using Mann–Whitney *U*-test and Spearman's correlation tests. *P* values <0.05 were considered statistically significant.

## 3 Results

### 3.1 Comparison of LAG-3 expression among the CHB, ASCs, and HCs groups

**Figure 1:**
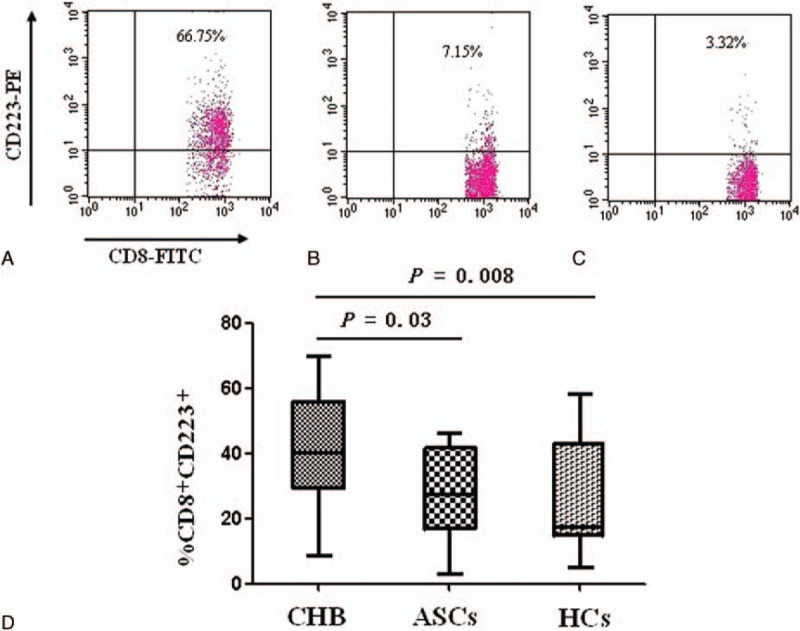
LAG-3 expression detected by flow cytometry among chronic HBV patients (CHB), chronic asymptomatic HBV carriers (ASCs), and normal control patients (HCs). The frequency of CD223-positive CD8^+^ lymphocytes in the CHB (A), ASC (B), and HCs (C). Panel (D) shows a comparison of the 3 groups (CHB: 41.15 ± 16.39%, n = 80; ASCs: 28.06 ± 13.85%, n = 10; HCs: 25.96 ± 16.27%, n = 18). LAG-3 = lymphocyte activation gene-3.

The frequency of CD223 expression in the CD8^+^ lymphocytes in the CHB group was significantly higher than in the HCs or ASCs groups (41.15 ± 16.39% vs. 25.96 ± 16.27%, *P* = 0.008 and 28.06 ± 13.85%, *P* = 0.03). The difference in CD223 expression frequency between the ASCs and HCs groups was not significant (28.06 ± 13.85% vs. 25.96 ± 16.27%, *P* = 0.65; Fig. 1).

### 3.2 LAG-3 expression in CD8^+^ T lymphocytes correlated with HBV DNA load and liver inflammation

Patients were divided into 4 subgroups based on serum HBV DNA and ALT levels. Group I, high ALT and low DNA (ALT ≥100 IU/L, DNA ≤5 log_10_ copies/mL, n = 14); Group II, high ALT and high DNA (ALT ≥100 IU/L, DNA >5 log_10_ copies/mL, n = 38); Group III, low ALT and low DNA (ALT <100 IU/L, DNA ≤5 log_10_ copies/mL, n = 25); and Group IV, low ALT and high DNA (ALT <100 IU/L, DNA >5 log_10_ copies/mL, n = 13).

**Figure 2:**
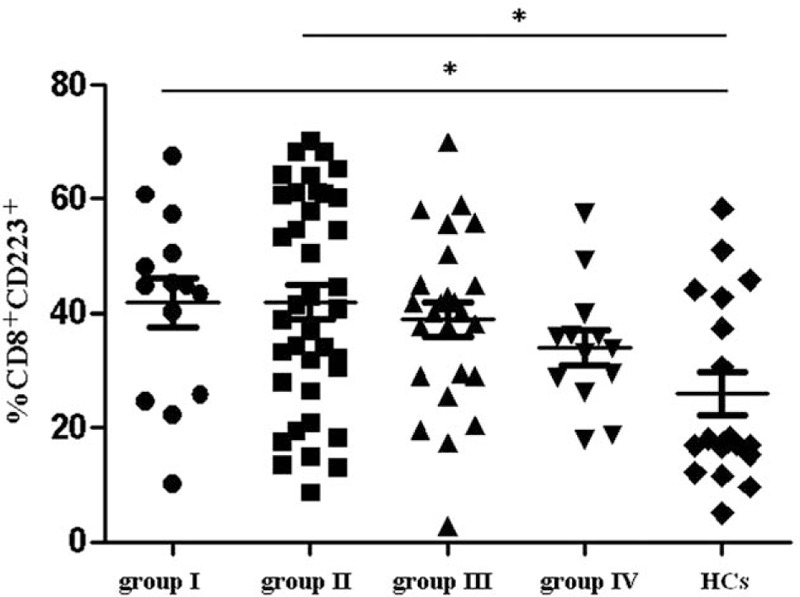
CD223-positive CD8^+^ T-lymphocyte was analyzed in patients with different HBV DNA and serum alanine aminotransferase (ALT) levels. Based on serum HBV DNA and ALT levels, 4 subgroups of patients were identified. Group I, high ALT and low DNA (n = 14); Group II, high ALT and high DNA (n = 38); Group III, low ALT and low DNA (n = 25); Group IV, low ALT and high DNA (n = 13). The frequency of CD223 expression in CD8^+^ lymphocytes was 41.79 ± 16.03% (Group I), 41.98 ± 18.63% (Group II), 38.99 ± 15.44% (Group III), and 34.09 ± 10.92% (Group IV). The difference among the 4 groups and the normal controls (HCs) was significant (^∗^*P* < 0.05).

The percentages of CD8^+^ T lymphocytes expressing CD223 among chronic hepatitis B patients with high ALT and low DNA (Group I) and high ALT and high DNA (Group II) were significantly higher than the percentage of CD8^+^ T lymphocytes expressing CD223 in the HCs group (41.79 ± 16.03% and 41.98 ± 18.63% vs. 25.96 ± 16.27%, *P* = 0.013 and *P* = 0.004, respectively). However, the percentages of CD223-positive CD8^+^ T lymphocytes among chronic hepatitis B patients with low ALT and low DNA (Group III) and low ALT and high DNA (Group IV) were not significantly different from the percentages observed in the HCs group (38.99 ± 15.44% and 34.09 ± 10.92% vs. 25.96 ± 16.27%, *P* = 0.051 and *P* = 0.097, respectively, Fig. 2).

**Figure 3:**
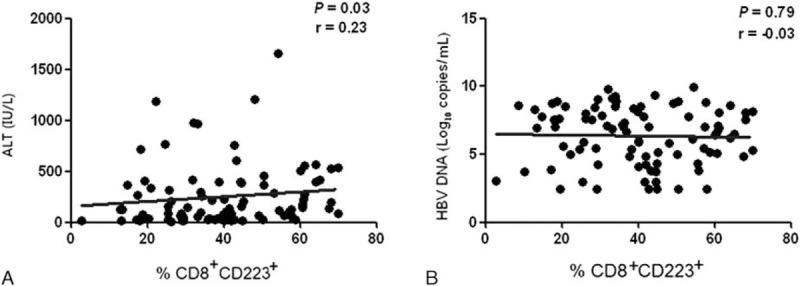
Correlations between CD8^+^ lymphocyte CD223 expression and the levels of HBV DNA and ALT. The correlation between CD8^+^ lymphocyte CD223 expression and (A) serum ALT levels (n = 90) and (B) HBV DNA load (n = 90). ALT = serum alanine aminotransferase, HBV = hepatitis B virus.

The CD223-positive CD8^+^ T-lymphocyte percentages in CHB patients and chronic HBV carriers positively correlated with ALT levels (*P* = 0.03, *r* = 0.23), but they did not correlate with HBV DNA levels (*P* = 0.79, *r* = −0.03; Fig. 3).

### 3.3 Differences in the number of IFN-γ-positive cells between CD8+CD223- and CD8+CD223+cells

**Figure 4:**
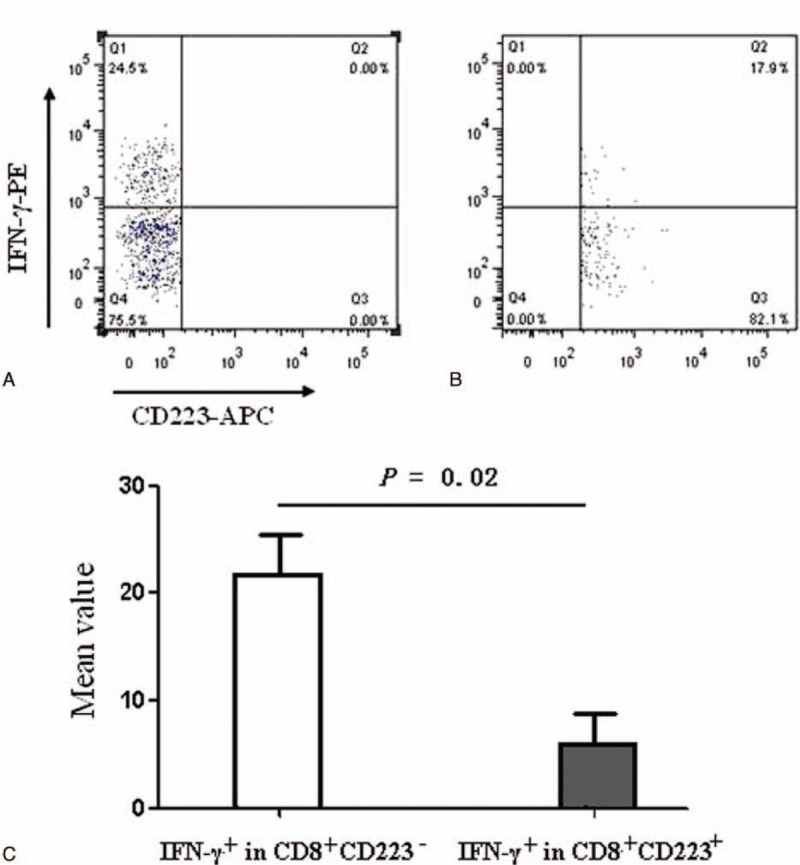
Differences in the percentages of IFN-γ-secreting cells between CD8^+^CD223^−^ and CD8^+^CD223^+^ cells. The percentages of IFN-γ^+^ cells that were CD8^+^CD223^−^ (A) or CD8^+^CD223^+^ (B). The frequency distributions of IFN-γ^+^ were significantly higher in CD8^+^CD223^−^ cells than in CD8^+^CD223^+^ cells isolated from PBMCs after treatment with a cell stimulation cocktail (21.70 ± 9.14% vs. 5.89 ± 6.24%, n = 25, *P* = 0.02) (C). IFN-γ = interferon-γ, PBMCs = peripheral blood mononuclear cells.

In CHB patients, the percentage of IFN-γ^+^ cells was significantly higher in CD8^+^CD223^−^ T cells than in CD8^+^CD223^+^ T cells (21.70 ± 9.14% vs. 5.89 ± 6.24%, *P* = 0.02; Fig. 4).

### 3.4 IFN-γ production was affected by LAG-3 antibody blockade

**Figure 5:**
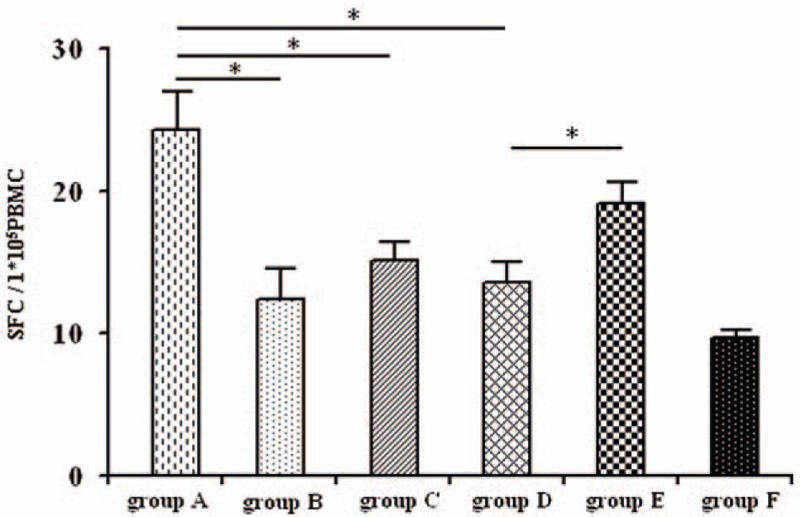
The effect of IFN-γ on lymphocyte production after binding LAG-3 with an LAG-3-specific antibody. Cells were divided into groups A to F as follows: Group A, HBcAg + CD223 antibody; Group B, HBcAg; Group C, HBcAg + IgG1; Group D, HBcAg + PD-L1; Group E, HBcAg + PD-L1 + CD223 antibody; Group F, autocrine group. Spot forming cells (SFC) per 1 × 10^5^ peripheral blood lymphocytes in Group A to F represented 24.00 ± 7.89, 12.63 ± 6.12, 15.38 ± 3.66, 13.67 ± 4.13, 19.38 ± 4.75, and 9.50 ± 0.84, respectively (n = 25). The significant differences were detected among those groups (^∗^*P* < 0.05). HBcAg = HBV core antigen IFN-γ = interferon-γ, LAG = lymphocyte activation gene-3.

IFN-γ production is frequently used as a marker of CD8^+^ cell function. IFN-γ production was significantly higher in cells isolated from CHB patients that were subsequently stimulated with HBcAg and CD223 antibody (Group A) when compared with cells stimulated with HBcAg alone (Group B; *P* = 0.006), HBcAg with isotype IgG1 (Group C; *P* = 0.006) and HBcAg with programmed death-ligand 1 (PD-L1) (Group D; *P* = 0.014). IFN-γ production was also higher in spot-forming cells stimulated with HBcAg, CD223 antibody and PD-L1 (Group E) when compared with cells stimulated by HBcAg and PD-L1 (Group D; *P* = 0.003). In contrast, there were no significant differences in IFN-γ production in the spot-forming cells when comparing stimulation with HBcAg and CD223 antibody and stimulation with HBcAg, CD223, and PD-L1 antibodies (*P* = 0.10; Fig. 5).

## 4 Discussion

This study investigated how the regulation of the immune response contributes to the balance between immunity strength and liver injury during the immune tolerant and active phases of chronic HBV infections. We approached this issue by detecting LAG-3 expression in CD8^+^ T cells and investigating the implications of the identified regulation.

We found that CD8^+^ T-cell expression of LAG-3 was significantly higher during the immune-active phase when compared with the immune tolerant phase and healthy control cells. Furthermore, IFN-γ production was significantly reduced in T cells with higher LAG-3 expression levels. The elevation of LAG-3 expression correlated positively with liver injury severity. Our results suggest that the functions of CD8^+^ T cells were suppressed by the expression of negative regulatory molecules during the immune-active phase. A reasonable explanation for this finding is that the strength of the immune response is regulated.

The immune-active phase is characterized by significant liver injury. Liver injury can help eliminate HBV from the infected liver when infected hepatocytes are destroyed by the immune response^[12]^; however, this mechanism of cell-killing based on HBV clearance can be fatal if a sufficiently large scale of liver injury is involved. An example of immune response-mediated liver injury is observed in fulminant hepatitis B, which is accompanied by a high mortality rate. The pathology of fulminant hepatitis B features widespread cell destruction due to a presumably intensified immune response to HBV-infected cells.^[13]^ The correct balance between a strong immune response and the scale of the resultant liver injury is required for a desirable outcome. This balance must primarily be achieved by regulating the strength of the immune response; as such, it appears that the immune response is regulated in chronic HBV infections.

In the current study, we found that the negative regulatory molecule LAG-3 was expressed at lower levels in CD8^+^ T cells when liver injury was minimal and that LAG-3 expression was increased to suppress CD8^+^ T-cell function when liver injury was prominent. Our results imply that T-cell suppression could be triggered by active liver injury, a process that involves many immune cells and cytokines.^[14]^ Therefore, cytokine elevation at injury sites could serve as a negative feedback mechanism that suppresses T-cell function and mitigates liver injury.

The direct impact of suppressing CD8^+^ T-cell function is 2-fold: suppression can limit the scale of liver injury, but it could also potentially prolong chronic HBV infection by slowing HBV clearance from the liver. It is a constant challenge to balance these 2 needs in HBV patients to ensure the desirable outcome of HBV clearance with minimal liver injury. Interventions targeting the mechanisms that control this balance would be of great help in the treatment of HBV patients once these mechanisms are better understood.

T-cell functions can be regulated by multiple inhibitory molecules, including PD-1, CD224, T-cell immunoglobulin mucin-3 (Tim-3), CTLA-4, and LAG-3.^[4,15]^ The inhibitory function of LAG-3 was recently described in chronic lymphocytic choriomeningitis virus infections in which high levels of LAG-3 and PD-1 expression were detected in exhausted CD8^+^ T cells.^[16,17]^ As suggested by the results of our current study, LAG-3 expression levels regulate the functions of CD8^+^ T cells during chronic HBV infections. T cells with LAG-3 expression exhibit reduced IFN-γ production.^[18]^ We found that CD8^+^ T-cell function was compromised by elevated LAG-3 expression, and this was demonstrated by the reduced number of IFN-γ producing cells that we observed in cells expressing high levels of LAG-3. However, compromised CD8^+^ T-cell function was restored when LAG-3 activity was inhibited by treatment with LAG-3 antibody. Furthermore, the functional restoration was more efficient when LAG-3 and PD-L1 antibodies were used when compared with PD-L1 alone. Studies have suggested that once LAG-3 molecules bind to MHC-II molecules on T cells, the resultant signals transmitted by the cytoplasmic domain of the KIEELE motif can inhibit T-cell proliferation and cytokine production (including IFN-γ; IL-2; tumor necrosis factor-α, TNF-a; and secretion from helper T cell 1, Th1). Because anti-LAG-3 antibodies are capable of preventing LAG-3 molecules from binding to MHC-II on T cells, LAG-3-induced suppression of CD4^+^ T and CD8^+^ T-cell function can be blocked.^[19–24]^ Matsuzaki et al^[24]^ found that the functional inhibition of T cells was stronger when LAG-3 and PD-1 were coexpressed than when PD-1 was expressed alone. High expression levels of LAG-3 and PD-1 in virus-specific CD8^+^ T lymphocytes restricted the antiviral responses of the CD8^+^ T cells. It was proposed that this was a mechanism responsible for maintaining a state of unresponsiveness to the viral infection. Thus, blocking both molecules should desuppress the immune cell population and improve antigen-specific CD8^+^ T-cell function.^[16,25]^ This finding could have therapeutic implications. For example, simultaneously blocking PD-1 and LAG-3 was shown to be a promising immunization approach for cancer treatment.^[26]^

In conclusion, the immune response in chronic HBV infection must be regulated to balance the strength of the response against the extent of the resultant liver injury. T-cell function was suppressed by elevated LAG-3 expression in patients with active liver injury. LAG-3-induced T-cell suppression was lower when liver injury was absent or minimal. Combined LAG-3- and PD-1-mediated suppression of T-cell function can be blocked using antibodies against both molecules. Our findings highlight the complexity of the mechanisms that regulate the immune response in chronic HBV infections, and our results could have therapeutic implications for future therapies that target the mechanisms suppressing T-cell function.
